# Floral Scent Evolution in the Genus *Jaborosa* (Solanaceae): Influence of Ecological and Environmental Factors

**DOI:** 10.3390/plants10081512

**Published:** 2021-07-23

**Authors:** Marcela Moré, Florencia Soteras, Ana C. Ibañez, Stefan Dötterl, Andrea A. Cocucci, Robert A. Raguso

**Affiliations:** 1 Laboratorio de Ecología Evolutiva y Biología Floral, Instituto Multidisciplinario de Biología Vegetal (CONICET-Universidad Nacional de Córdoba), Córdoba CP 5000, Argentina; fsoteras@imbiv.unc.edu.ar (F.S.); acibanez@imbiv.unc.edu.ar (A.C.I.); aacocucci@imbiv.unc.edu.ar (A.A.C.); 2 Department of Biosciences, Paris-Lodron-University of Salzburg, 5020 Salzburg, Austria; stefan.doetterl@sbg.ac.at; 3 Department of Neurobiology and Behavior, Cornell University, Ithaca, NY 14853, USA

**Keywords:** brood-site deceptive flowers, ecological niche modelling, fly pollination, flower scent, hawkmoth pollination, nightshades, pollinator shift, South America

## Abstract

Floral scent is a key communication channel between plants and pollinators. However, the contributions of environment and phylogeny to floral scent composition remain poorly understood. In this study, we characterized interspecific variation of floral scent composition in the genus *Jaborosa* Juss. (Solanaceae) and, using an ecological niche modelling approach (ENM), we assessed the environmental variables that exerted the strongest influence on floral scent variation, taking into account pollination mode and phylogenetic relationships. Our results indicate that two major evolutionary themes have emerged: (i) a ‘warm Lowland Subtropical nectar-rewarding clade’ with large white hawkmoth pollinated flowers that emit fragrances dominated by oxygenated aromatic or sesquiterpenoid volatiles, and (ii) a ‘cool-temperate brood-deceptive clade’ of largely fly-pollinated species found at high altitudes (Andes) or latitudes (Patagonian Steppe) that emit foul odors including cresol, indole and sulfuric volatiles. The joint consideration of floral scent profiles, pollination mode, and geoclimatic context helped us to disentangle the factors that shaped floral scent evolution across “pollinator climates” (geographic differences in pollinator abundance or preference). Our findings suggest that the ability of plants in the genus *Jaborosa* to colonize newly formed habitats during Andean orogeny was associated with striking transitions in flower scent composition that trigger specific odor-driven behaviors in nocturnal hawkmoths and saprophilous fly pollinators.

## 1. Introduction

Chemical communication via floral scent plays a central role in attracting pollinators and is considered a key innovation promoting pollinator shifts and diversification in flowering plants [1,2,3]. Floral scent combines with non-chemical components of floral display in complex, multi-sensory communication between flowers and their animal visitors [4,5,6]. Nevertheless, floral scent itself can be considered as a multichannel signal, and small evolutionary changes in scent composition may open (or close) signal channels for certain flower visitors that directly affect plant fitness [7,8,9]. Thus, pollinator olfactory sensory acuity and olfactory learning are important components of the selective environment that shape floral evolution in angiosperms [10,11,12].

Flowering plant lineages are quite labile in their scent chemistry in ways that are linked with their evolutionary success [13,14]. Although some scent compounds are restricted to particular lineages [15,16], scent composition may be highly variable within a genus [17,18,19] or even within a species [20]. The amount of scent emission as well as its chemical composition could change through adaptation to divergent pollinator environments [21] or mating systems [22,23], the presence of floral enemies [24] or the combined influence of pollinators and herbivores [25]. Thus, scent composition often tracks floral diversification related to shifts in pollination systems and other aspects of floral function [11,26,27].

An important aspect in considering the relevance of pollinators as drivers of floral diversification is the context in which diversification is promoted. For example, floral diversification may occur when plants colonize new geographical areas where the local pollinator fauna is depauperate or differs in relation to the source area [28,29]. Coastal and inland populations of *Diplacus aurantiacus* (Curtis) Jeps. (Phrymaceae) in southern California, USA, differ in flower color and primary pollinator [30]. Reciprocal transplant experiments showed that hummingbirds preferred red-flowered, coastal plants in both habitats, whereas hawkmoths exclusively visited yellow-flowered, inland plants but only at inland sites [31]. The persistence of floral color differences across a geographic cline, in the face of gene flow between inland and coastal populations, suggests that color differences in *D. aurantiacus* are adaptive [32].

Studies of floral scent generally assume that phenotypic differences reflect heritable adaptive changes due to pollinator-mediated selection. For example, there are region-specific differences in phenotypic selection on floral scent in *Gymnadenia odoratissima* (L.) Rich. orchids and *Arum maculatum* L. aroids associated with different pollinator assemblages at different altitudes in Switzerland [33] and regions across the Alps in Europe [34], respectively. The inference of heritable adaptive variation in scent was supported by an experimental evolution study using rapid-cycling *Brassica rapa* L. plants, in which floral scent composition diverged in response to the selective pressures exerted by different pollinator assemblages, in the absence of environmental variation [21]. However, floral scent variation may also be influenced by abiotic environmental factors, such as soil chemistry, ozone or drought [35,36,37]. Drought may directly affect scent emission in real time due to phenotypic plasticity, with associated effects on pollinator attraction and behavior [38]. Similarly, temperature and photoperiod shape diel floral emission patterns [39,40] as well as pollinator flight periods [41]. We expect these environmental factors to combine across altitudes and latitudes, creating local differences in “pollinator climate” *sensu* Grant and Grant [42], such as moth pollination limited by evening temperatures at higher altitude [43]. Thus, diversification of floral scent at larger taxonomic scales (e.g., across clades or genera), especially in lineages that occupy diverse geographical regions, is likely to reflect divergent selective pressures exerted by variable pollinator climates [44,45].

In this context, the Andes of South America provide a unique geographic scenario under which several pollinator transitions have been recognized. The Andean uplift created isolated “sky islands” surrounded by drier valleys which act as barriers to the dispersal of species [46]. On the other hand, the climate change of the Pleistocene caused repetitive range expansion and contraction of species distribution, allowing down-slope migration through lowland elevation barriers such as valleys [47]. These scenarios of allopatric and clinal speciation are clearly relevant for explaining the remarkable concentration of plant radiations in the high-elevation Andes [48,49,50,51]. Divergence can result from selection by the environment or through interactions with other organisms, or even through stochastic processes (e.g., genetic drift) that result in phenotypic changes which may or may not be adaptive [52,53,54].

In particular, we considered that the nightshade genus *Jaborosa* Juss., with two clades showing different ecological histories and contrasting pollination environments (hawkmoths, saprophilous flies and generalized small insects [55,56]), presents an opportunity to explore the evolution of floral scent under novel pollinator climates. The aim of this study was to chemically characterize floral scent in the two clades of *Jaborosa*, that have different geographic affinities: the “Subtropical Lowland clade” includes species inhabiting lowland areas, and the “Andean clade” includes species mainly distributed across the Andes mountain range and Patagonian steppe of temperate South America. A previous study suggested that the ability of *Jaborosa* species to colonize newly formed environments during Andean orogeny and its subsequent ecological changes were concomitant with transitions in flower color, as perceived by different pollinator functional groups [56]. Here, we asked whether *Jaborosa* species with different pollination modes differ predictably in floral scent profiles, taking into account the evolutionary and geoclimatic context of diversification across the genus. Our previous studies of *Jaborosa* have addressed functional aspects of floral scent in focal case studies [57,58], phylogenetic reconstruction [55] and broader genus-wide trends in flower color as perceived by pollinators [56]. Here, we have used an ecological niche modelling (ENM) approach to analyze which environmental variable(s) (e.g., latitude, elevation, temperature, and precipitation) have exerted the strongest influence on floral scent profiles across a broader sample (eight spp.) of the genus. Given the geographic distribution of extant *Jaborosa* species, we expected that the chemical composition of floral scent would reflect shifts in pollinator climate from hawkmoth-dominated subtropical lowlands to fly-dominated high altitudes (Andes) or latitudes (Patagonian Steppe). We predicted that nonrandom combinations of flower scent and pollination mode, reflecting pollinator-specific olfactory preferences and supported by earlier focal studies [57,58], should play out across our larger genus-wide sample, spanning different geographical domains. This prediction stems from previous studies, in which the experimental addition of volatile sulfides to flowers of *Jaborosa integrifolia* Lam., a lowland species pollinated by hawkmoths, resulted in the attraction of saprophilous flies, which do not normally visit these flowers. These results provided evidence that the emission of fetid floral volatile organic compounds (VOCs) is sufficient to attract saprophilous flies to flowers, largely irrespective of other floral features and geographic region [58], as has been demonstrated in similar studies in Africa [26], Europe [59] and Asia [60].

Available data lead us to predict a clear contrast in scent composition between the primary pollination systems in *Jaborosa* [61]. Flowers pollinated by saprophilous flies emit fetid odors recalling feces, rotting meat, or carcasses. These floral scents are usually characterized by the presence of low molecular weight VOCs resulting from protein decay, with volatile sulfides (e.g., dimethyl disulfide, dimethyl trisulfide) characteristic of carrion or carnivore dung, phenol, cresols and indole indicative of herbivore dung, and aliphatic acids, ketones, amines and other compounds common to urine [62,63,64]. Although such odors are perceived by humans as “strong” due to their nauseous quality, they are seldom recorded in large amounts, suggesting that flies are highly sensitive to their presence [59]. In contrast, night-blooming flowers pollinated by hawkmoths often emit powerful perfume-like scents characteristic of jasmines, gardenias and jonquils [65], shown to elicit upwind flight and attraction by moths, presumably from a distance [66]. Acyclic, oxygenated terpenes and aromatic VOCs are ubiquitous scent components of hawkmoth-pollinated flowers, along with nitrogenous aldoximes and/or indole [13,15,65,67]. Several studies have demonstrated that hawkmoths show antennal sensitivity to terpene alcohols (e.g., linalool), aromatic alcohols, aldehydes and esters [68,69,70], and behavioral responses to scent blends containing them [49,71,72]. Thus, our analyses of floral scent across the genus *Jaborosa* were performed with clear expectations of distinct chemical composition associated with different pollinator climates and geographic distributions.

## 2. Results

### 2.1. Ecological Niche Modelling of JABOROSA in Geographical Space

**Figure 1:**
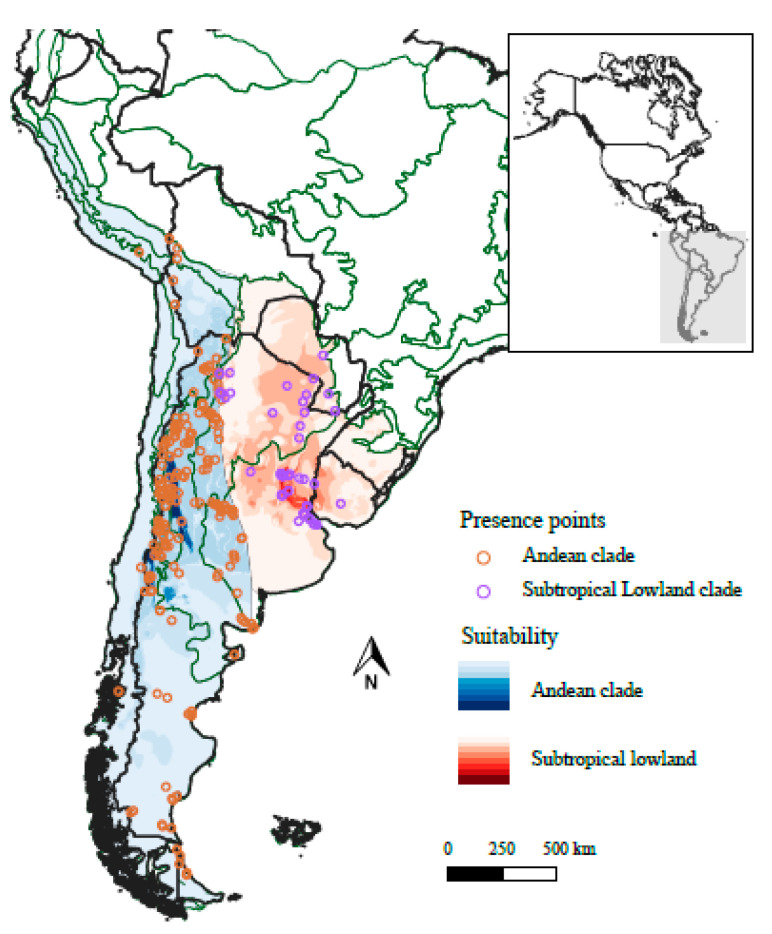
Environmental suitability and occurrence map of the nightshade genus *Jaborosa* Juss. The darkest color shades represent the areas with the highest suitability values for the Andean (blue) and Subtropical Lowland (red) clades. Phytogeographical regions (green lines) correspond to those provided by [73]. National boundaries delimited with black lines were obtained from [74].

The areas of the highest climatic suitability for the Andean clade comprised the phytogeographic Andean and Monte regions; and for the Subtropical Lowland clade the Pampean Province (Figure 1). Four of the eight selected bioclimatic variables, mostly related to temperature and humidity, contributed 94% to the ENMs of the Andean Clade; while two of the six selected variables, altitude and specific humidity seasonality, contributed 90% to the ENMs of the Subtropical Lowland clade (Table S1). The two first axes of the environmental principal component analysis (PCA) explained 78% of total variance (PC1 = 55% and PC2 = 23%). Four environmental variables were highly associated with PC1 scores: one positively (altitude (40%)), and three negatively (mean temperature of the least humid quarter, maximum temperature of the warmest month, and specific humidity seasonality (53%, 51%, and 48%, respectively)). Two variables were associated with PC2 scores: temperature seasonality and mean diurnal range temperature (67 and 69%, respectively).

### 2.2. Composition and Variation of Floral Scent among Jaborosa Species

Floral scent in the genus *Jaborosa* is chemically complex, representing diverse biosynthetic pathways and reflecting substantial differences in emission strength. Accordingly, we used a combination of methods to collect and identify the VOCs present in the floral scent of the eight sampled *Jaborosa* species. Dynamic headspace-GC-MS analyses of solvent-eluted samples led to the identification of 26 VOCs emitted by the night-blooming flowers of *Jaborosa runcinata* Lam. and *J. integrifolia* (Table S2) during the first hour of anthesis, when hawkmoth visitation generally is most frequent. In contrast, this method was insufficient to characterize VOCs from day-blooming flowers of *Jaborosa rotacea* (Lillo) Hunz. and Barboza and one of two replicates of *Jaborosa leucotricha* (Speg.) Hunz., revealing only trace amounts of 2-heptanone and p-methylanisole in the other replicate of *J. leucotricha*. Similarly, expanding dynamic headspace collection to 3 h led to the detection of trace amounts of only 11 VOCs from full inflorescences (15 flowers) of *Jaborosa sativa* (Miers) Hunz. and Barboza, a poor representation of the actual floral chemistry of this species (see Tables S3 and S4). 

The scent blend of *J. runcinata* was simple, dominated by benzaldehyde (mean ± s.e. of 92 ± 5% of total emissions) and structurally related aromatic compounds (benzyl alcohol, benzyl benzoate, benzyl salicylate). These same compounds were present in the bouquet of *J. integrifolia*, albeit in different relative proportions, along with a more complex blend of aromatic esters (methyl benzoate, methyl salicylate), oxygenated terpenoids (linalool, farnesol-related VOCs) and long-chain aliphatic aldehydes (Table S2). Chemical composition was consistent among samples collected from wild vs. greenhouse cultivated plants of *J. integrifolia* across three years, whereas their relative emission rates per flower were roughly four-fold higher in cultivation than in natural settings (Table S2), potentially due to evening temperatures or other environmental factors. 

Direct thermal desorption-GC-MS solid-phase extraction (SPE) analyses were more effective in the collection and analysis of floral volatiles from *Jaborosa* species of the Andean clade that grow in remote natural settings. For example, this method doubled the number of VOCs (25) detectable from flowers of *J. sativa*. A total of 84 VOCs were identified from trapped floral headspace of the eight studied species (Table S3). Floral VOCs from these species included 25 isoprenoid compounds (16 monoterpenes, 8 sesquiterpenes, 3 irregular terpenoids), 21 aromatic compounds, 18 aliphatic compounds (esters, ketones, alcohols, aldehydes and alkenes), 11 ringed nitrogenous compounds (pyrroles, piperidines and indole) and 7 sulfurous compounds (thioesters, oligosulfides and a thiophene; Table S3). Floral scent samples collected from two species of the Subtropical Lowland clade, *J. integrifolia* and *J. runcinata*, recovered nearly all of the compounds identified from solvent samples (Table S2), demonstrating internal consistency among methods for large, hawkmoth-pollinated flowers. Floral scent of the third species of the Subtropical Lowland clade, *Jaborosa odonelliana* Hunz., was a simple blend dominated by acyclic sesquiterpene alcohols. Our data also indicate high interspecific variation in floral scent composition among the species of the Andean clade. Floral scent was a binary blend of oligosulfides in *J. rotacea*, whereas the remaining taxa showed complex, often species-specific blends dominated by thioesters and short-chain aliphatic esters in *J. sativa*, monoterpenes and nitrogenous ringed compounds in *Jaborosa laciniata*, p-cresol and rare, monoterpene derivatives in *J. leucotricha,* and more conventional terpenoids and aromatic volatiles in *Jaborosa reflexa* Phil. (Table S3).

Finally, an intensive focal study using solid-phase micro extraction (SPME) gas chromatography–mass spectrometry (GC-MS) analysis led to the identification of 57 VOCs emitted by dehiscent flowers of the saprophilous fly-pollinated species *J. sativa* (Table S4). Pollen-mature flowers emitted a complex floral scent that included several S-volatiles with mass spectra suggesting a series of thioesters, only a few of which were detected in the other *Jaborosa* species. These findings are consistent with our perception of garlic notes, along with fetid aromatic compounds (cresols) and nitrogenous compounds (indole) reminiscent of feces. Floral scent composition did not vary when incrementing the number of flowers of dehiscent flowers (4, 8 and 16). Fewer VOCs were detected from indehiscent flowers and buds (Figure S1). Floral scent composition did vary among flower parts in dissected flowers, with petals emitting most of the floral VOCs present in whole, mature flowers. Only a few additional VOCs were detected from dissected fertile organs (anthers and stigma) and composition varied among samples (Figure S1).

### 2.3. Evolution of Floral Scent in JABOROSA and Its Association with Pollination Mode and Climate

**Figure 2:**
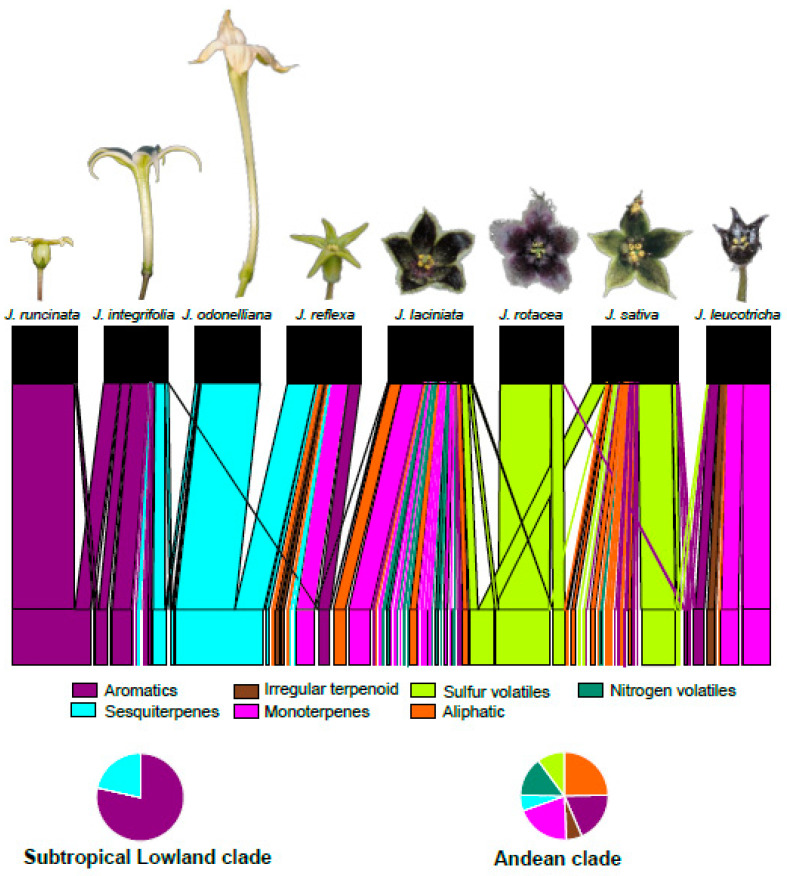
Floral Volatile Organic Compounds (VOCs) emitted by the studied *Jaborosa* species. Plant –floral volatile network showing the plant species (upper rectangles) connected to emitted floral VOCs (bottom rectangles). Bar widths correspond to the relative percentage of VOCs, i.e., all compounds present in the floral scent of a given *Jaborosa* species add up 100%. Colors show different chemical classes. Floral VOCs are listed in Supplementary Table S3.

Chemical complexity and compound diversity of floral scent varied markedly between *Jaborosa* clades. Two chemical classes (aromatics and sesquiterpenes) dominated the volatile blends produced by the three hawkmoth-pollinated *Jaborosa* species from the Subtropical Lowland clade. In contrast, six chemical classes (aliphatics, aromatics, irregular terpenoids, monoterpenes, sesquiterpenes, nitrogenous and sulfurous compounds) were represented in the floral scent of the five *Jaborosa* species (four pollinated by saprophilous flies and one with generalized pollination) sampled from the Andean clade (Figure 2). In addition to this clear association between VOCs’ chemical classes and clades, our data also revealed high variation in chemical composition among species, with most of the floral VOCs being species-specific (Figure 2, Table S3). These trends were amplified by the presence of metabolic series or “runs” of unique, related compounds, such as six thioesters in *J. sativa* (Table S4) and seven piperidine-related N-volatiles in *J. laciniata* (Table S3; Figure S2). A detailed summary of the taxonomy of short chain esters found in *J. sativa* is shown in Table S3.

In the Subtropical Lowland clade, the scent blends of *J. odonelliana* and *J. integrifolia*, which replace each other ecologically from east to west in northern Argentina (see Figure 1 in ref. [56]), shared oxygenated sesquiterpenoids ((E)-nerolidol, isomers of farnesol and (E,E)-farnesal), whereas the floral scent of partially sympatric *J. integrifolia* and *J. runcinata* overlapped in a few aromatic compounds (benzyl alcohol, benzaldehyde, benzyl benzoate), with additional related compounds (methyl benzoate, methyl salicylate) unique to the more complex blend of *J. integrifolia*. Available evidence indicates that these three species are pollinated infrequently by large nocturnal moths [56].

**Figure 3:**
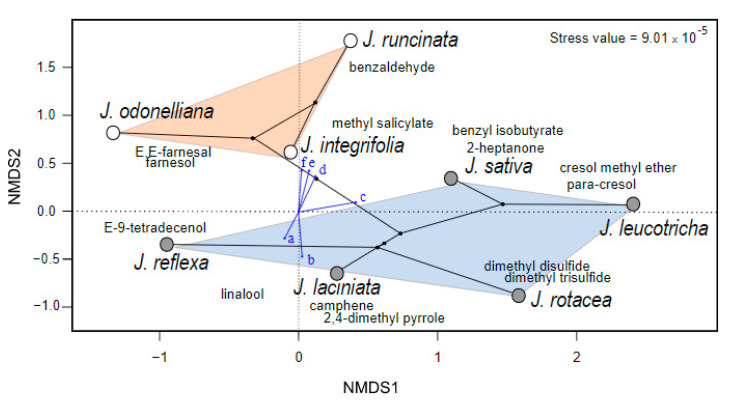
Phylo-scent space of studied *Jaborosa* species. Non-metric multidimensional scaling (NMDS) of the floral scent profiles (Supplementary Table S3). Phylogenetic relationships among *Jaborosa* species were projected into the space (white and grey circles show species from the Subtropical Lowland and Andean clades, respectively). Some VOCs that are important explaining differences between floral scent samples are shown. Blue arrows show the influence of environmental variables in scent space (a: temperature seasonality, b: altitude, c: mean diurnal range temperature, d: maximum temperature of the warmest month, e: specific humidity of the most humid month, and f: mean temperature of the least humid quarter). According to a PERMANOVA analysis, the scents differed between species of the Subtropical and the Andean clades.

Interestingly, some of the same VOCs that typify hawkmoth pollination in lowland *Jaborosa*, such as (E)-nerolidol and related terpenoid hydrocarbons (linalool, (E)-β-caryophyllene, (E,E)-α- and (E)-β-farnesene, (E)-4,8-dimethyl-1,3,7-nonatriene, (E)-4,8,12-trimethyl trideca-1,3,7,11-tetraene) were detected consistently in the floral headspace of *J. reflexa*, a species from the Andean clade, which shows generalized pollination by small insects (Figure 2 and Figure 3, Table S4), as well as population-specific variation for overall scent composition (Table S3, Figure S3). The remaining four *Jaborosa* species from the Andean clade are pollinated by saprophilous flies and produce VOCs not observed in the Subtropical Lowland clade. The scent blends of three species (*J. rotacea*, *J. sativa* and *J. laciniata*) shared the presence of dimethyl disulfide (DMDS) and dimethyl trisulfide (DMTS), universal indicators of carrion mimicry, whereas a fourth species (*J. leucotricha*) lacked these sulfides but emitted other compounds (p-cresol, β-citronellene) indicative of herbivore fecal mimicry (Tables S3 and S5). 

The phylo-scent-space showed that the two *Jaborosa* clades differed significantly in floral scent chemical composition (Figure 3, Figure S3). Six environmental variables that contributed the most to the ENMs of the two clades helped to explain the differences observed in scent profiles. Maximum temperature of the warmest month and humidity were associated with the samples from the Subtropical Lowland clade, whereas temperature seasonality, altitude and diurnal range temperature were associated with the samples from the Andean clade (Figure 3, Table S1).

**Figure 4:**
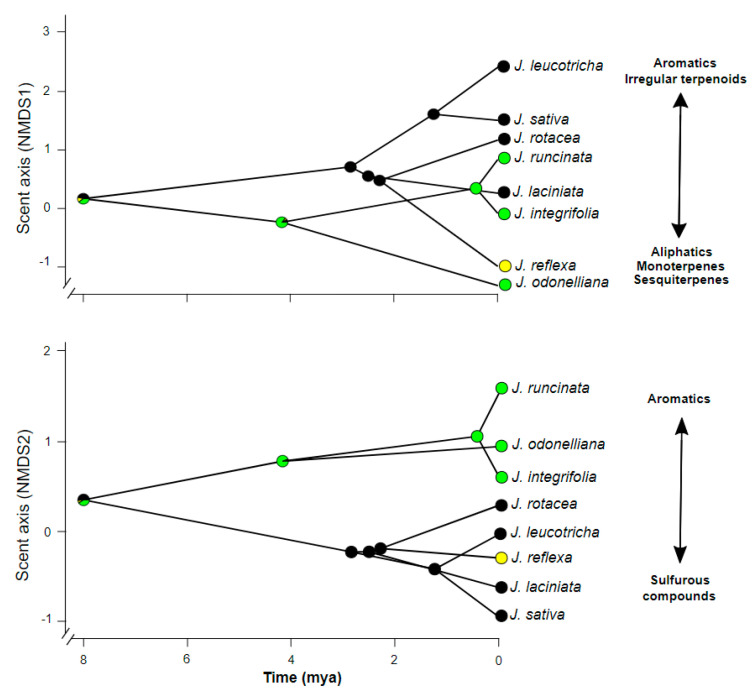
Reconstruction of floral scent and pollination mode in *Jaborosa* Juss. Phylogenetic relationships among the eight *Jaborosa* species were projected in the space defined by floral scent profile; y-axis: NMDS1 (above) and NMDS2 (below) scores from Figure 3; x-axis: divergence time. Pie charts on each node show the posterior probability of each pollination mode (nocturnal hawkmoths in green, generalist by small insects in yellow and saprophilous flies in black) retrieved by 100 stochastic character mappings (see text for further details). Chemical classes of the floral VOCs that are important explaining the ordination are shown on the right.

**Figure 5:**
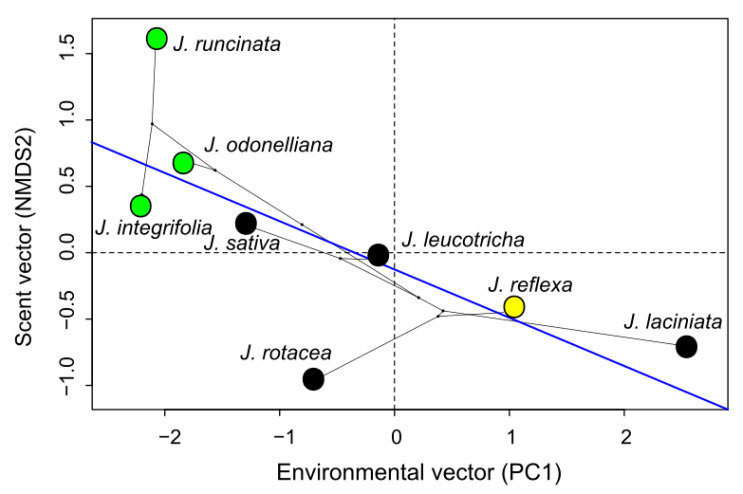
Evolutionary transitions along the scent axis in *Jaborosa* Juss. Phylogenetic generalized least squares (PGLS) correlation between environmental suitability and scent profiles. Blue line represents the slope. Different colors show pollination modes: green for hawkmoths, black for saprophilous flies and yellow for generalized small insects.

Diversification in *Jaborosa* across southern South America was associated with a pollinator shift from nocturnal hawkmoths in subtropical lowlands to saprophilous flies in the high elevation areas and occurred in concert with changes in floral scent chemical composition. Ancestral reconstructions of pollination modes were contingent with changes in the floral scent profiles (Figure 4). These changes in floral scent composition varied from relatively simple profiles dominated by aromatics and sesquiterpenes in the hawkmoth-pollinated species to complex VOC mixtures including sulfurous, ringed nitrogenous, monoterpene, aliphatic and irregular terpenoid compounds in the saprophilous fly-pollinated species (Figure 4). The most likely recent common ancestor of the extant species of the Subtropical Lowland clade (about 4.2 Ma) was reconstructed as being pollinated by hawkmoths, while the most likely recent common ancestor of the extant species of the Andean clade (about 2.8 Ma) was reconstructed as being pollinated by saprophilous flies. Generalized pollination in *J. reflexa* appears to have evolved more recently from a specialized saprophilous fly-pollinated ancestor (Figure 4). Interestingly, the environmental niche of *J. reflexa* significantly differed from *J. laciniata*, and partially overlapped with *J. leucotricha* and *J. volkmannii*, the other three species that totally of partially overlap in their geographical distribution (Figure S4). Environmental suitability (PC1 scores summarizing the six climatic variables that largely explained the ENMs) had a significant impact on the floral scent profiles (scent axes from NMDS biplot) while controlling for potential phylogenetic signal (i.e., non-independence of the residuals; F = 6.86, *p* = 0.0396; Figure 5, Table S6).

## 3. Discussion

### 3.1. Methodological Challenges

We were motivated to study the evolution of floral scent in the genus *Jaborosa* because the two primary modes of pollination are so utterly different (i.e., brood site deception of saprophilous flies vs. honest nectar-rewarding pollination by hawkmoths), as are the geographic distributions associated with distinct pollinator climates [56]. To achieve our goal, we surmounted several challenges. First, it was not possible to design a common garden study to collect scent samples from all target species because of factors including harsh climate, restricted access to remote natural populations and the inherent difficulty of cultivating *Jaborosa* species of the Andean clade under greenhouse conditions. Thus, with a few exceptions, most of our scent data were sampled opportunistically from wild plants growing in remote settings. Second, the flowers of most *Jaborosa* species smelled strongly to our human noses for entirely different reasons; hawkmoth-pollinated flowers of the Subtropical Lowland clade emit large amounts of common and inoffensive scent compounds, whereas fly-pollinated flowers of the Andean clade emit low amounts of pungent, fetid compounds to which the human nose is highly sensitive. This distinction, which highlights key differences in the chemical structures and properties of the VOCs associated with hawkmoth vs. fly pollination, compelled us to utilize three different methods (i.e., dynamic headspace desorbed with solvent, SPE-direct thermal desorption, and SPME fibers) in order to ensure that we could detect specific compounds across our sampled species. In fact, we often performed “dose-response” experiments in which we systematically increased volatile equilibration or collection times in remote field settings, to ensure that floral VOCs could be detected in at least some replicates when GC-MS analyses were performed, weeks to months later, in our respective laboratories. Despite such efforts, we failed to detect floral VOCs from the foul-scented, saprophilous fly-pollinated *Jaborosa magellanica* (Griseb.) Phil.

### 3.2. Clade-Specific Volatile Patterns

We detected a clear differentiation of floral scent among clades and species within *Jaborosa*, associated with particular floral syndromes previously reported in the literature [61]: S-volatiles (carrion mimicry; [75,76,77,78]), aliphatic alcohols/esters (fermentation; [79]), aromatic alcohols/N-volatiles (fecal mimicry; [62]), aromatic alcohols/esters (reward-based pollination, including sphingophily; [71,80]), isoprenoid hydrocarbons (generalized pollination; [1]), and isoprenoid alcohols (reward-based pollination, especially sphingophily; [15,70].

The floral scents of hawkmoth pollinated *Jaborosa* species (*J. integrifolia*, *J. odonelliana* and *J. runcinata*) were chemically simple, featuring large amounts of relatively few compounds (i.e., oxygenated sesquiterpenes or aromatic volatiles) shown to be nearly ubiquitous in sphingophilous flowers in temperate South America [13,49,67,71,81] and elsewhere [15,65]. Interestingly, scent composition among sphingophilous *Jaborosa* species from the Subtropical lowland clade was divergent rather than conservative, as volatile emissions were dominated by methyl salicylate in *J. integrifolia*, benzaldehyde in *J. runcinata* and (E)-nerolidol in *J. odonelliana* (Tables S1 and S2). This trend reflects larger patterns among congeneric hawkmoth-pollinated species in the Nyctaginaceae [82], Cactaceae [49] and elsewhere in the Solanaceae (genus *Nicotiana* L.; [13]): loosely convergent evolution of certain volatile classes nested within species-specific blend composition. Behavioral assays performed with *Manduca sexta* (Linnaeus, 1763), a widespread species thought to be representative of the hawkmoth guild present in lowland warm-temperate Argentina [83], confirm that a variety of volatile terpenes, aromatics and N-compounds attract these moths to flowers [49,72,84,85,86]. These findings indicate that convergent evolution is not constrained to a narrow sensory bias for *M. sexta* and ecologically similar hawkmoths, and suggest that species-specific blends may be learned by moths to promote floral constancy [87,88].

In parallel, the four saprophilous fly-pollinated species (*J. leucotricha*, *J. laciniata*, *J. rotacea* and *J. sativa*) generally were characterized by the presence of universal volatile components (e.g., oligosulfides, cresol, indole) indicative of carrion- or dung-mimicking flowers, fungi and mosses [62,63,64,89]. Specifically, most of the Andean clade species emitted DMDS and DMTS, behaviorally active compounds that are common to rotting meat and carrion-mimicking flowers worldwide [26,59,60]. However, beyond these core volatiles, composition of the full scent bouquets among these taxa was divergent, dominated by oligosulfides in *J. rotacea*, thioesters and short, branched chain aliphatic esters in *J. sativa*, monoterpenes and ringed N-compounds in *J. laciniata,* and cresols and saturated monoterpenes in *J. leucotricha*.

### 3.3. Niche Dimensions within Sapromyophily: A Closer Look at Scent in Fly-Pollinated Jaborosa Species

A landmark survey [62] of floral scent variation in fly-pollinated stapelioid milkweeds across southern Africa challenged the notion that sapromyophilous plants use the same, fetid volatiles to attract flies to brood-site deceptive flowers, as it described unexpected variation in compound identity, blend complexity and associated differences in floral color and shape. A more focused analysis of carrion mimicry in the South African orchid *Satyrium pumilum* Thunb. revealed that variation in flower size and oligosulfide concentration was associated with niche partitioning between sarcophagid and calliphorid flies, which appear to specialize in dead animals of different sizes [90]. Subsequent studies in the same region revealed that flies attracted by scent are filtered as pollinators by non-chemical traits that either restrict access by size (floral guardrails [91]) or encourage landing by simulating the presence of other flies (tepal spots [92]).

Similar niche partitioning may be occurring among fly-pollinated Andean *Jaborosa* species, given observed variation in flower size and morphology, scent composition and the diversity of observed fly visitors [56]. Here we examined the floral scent composition of Andean *Jaborosa* species for hints of niche dimensionality, by virtue of supplemental GC-MS analyses of cut flowers in smaller headspace chambers. Greenhouse cultivated plants of *J. rotacea* and *J. sativa* allowed us to use SPME-GC-MS to analyze scent from whole and dissected flowers. For *J. rotacea*, the oligosulfides that dominated samples analyzed via thermal desorption (Table S3) were accompanied by other compounds (acetophenone, small aliphatic alcohols, aldehydes and ketones) associated with dehiscing anthers, suggesting pollen as a source tissue [57]. These compounds are characteristic of urine, feces or decaying substances [53,54], suggesting a more complex brood-site model than rotting meat [93]. For *J. sativa*, SPME-GC-MS revealed a complex series of thioesters formed with C2–C7 acids (Figure S2), one of which was present, along with an aromatic S-compound (2-isopentyl thiophene), in samples collected from unopened flower buds (Table S4). Thioester formation, through esterification of carboxylic acids with methionol, is known to be mediated by cheese-making bacteria such as *Brevibacterium linens* [94]. It is unclear whether floral microbes are responsible for thioester biosynthesis in flowers of *J. sativa*. Thioesters are exceedingly rare in floral scent studies, having only been identified (S-methyl thiolacetate) from bat-pollinated flowers of *Cephalocereus* Pfeiff. cacti [95]. Apart from thioesters, the core *J. sativa* floral bouquet recovered by all headspace-GC-MS methods used (Tables S3 and S4) included compounds (oligosulfides, indole, p-cresol, 2-ketones and C6–C9 aliphatic aldehydes) that predict a more generalized spectrum of floral visitors, including flies attracted to carrion, dung, hides and urine. This agrees with our field observations that a broad spectrum of saprophilous flies visited the flowers [56]. A total of five “morpho-species” from three families (Calliphoridae, Muscidae and Sarcophagidae) pollinated *J. sativa* flowers.

The remaining two fly-pollinated species in our study could not be cultivated, so we performed dose–response sampling with SPE micro traps in remote Andean field sites. In flowers of *J. laciniata*, the presence of oligosulfides was complemented by generic monoterpenes and a metabolic series of ringed N-compounds with mass spectra suggesting pyrroles and piperidines (Table S3, Figure S2). Common monoterpenes (e.g., terpinolene, linalool) are known to attract flies (e.g., *Musca domestica* Linnaeus, 1758) that otherwise favor fetid odorants, such as indole [96]. High floral emissions of generic monoterpenes, combined with low emissions of DMDS and DMTS, may explain why a different spectrum of flies (fewer calliphorids, more sarcophagids and novel tachinids) is attracted to flowers of *J. laciniata* than to oligosulfide baits in Andean field sites [58]. Similar to thioesters, pyrroles and piperidines are extremely rare in floral scents [1], with similar volatiles identified only recently from herbivore dung-mimicking inflorescences of *Arum maculatum* [97] and from food-rewarding flowers of European pear (*Pyrus communis* L.; [98]). Both taxa are attractive to flies, and the relevance of these uncommon volatiles to fly pollination remains to be tested. When floral samples of *J. laciniata* were equilibrated overnight, their scent composition shifted to include aliphatic and aromatic alcohols and ketones along with one thioester produced by flowers of *J. sativa* (Tables S3 and S4). These patterns were not evident in a whole-plant sample, which was dominated by the monoterpene-rich scent of the foliage [58].

In contrast, the nauseating floral scent of *J. leucotricha* is noteworthy for its absence of volatile oligosulfides, instead consisting of p-cresol (and its methyl ether), aliphatic ketones and hydrated monoterpenes (3,7-dimethyl-1,6-octadiene [=β-citronellene], 3,7-dimethyl-1-octene) common to plants that mimic herbivore dung as a brood site, such as *Arum maculatum* [64,97,99,100], stapelioid milkweeds [62] and *Splachnum* L. ex Hedw. mosses [100]. The fly assemblage visiting *J. leucotricha* was narrower in comparison to the other three saprophilous fly-pollinated *Jaborosa* species. The flies pollinating *J. leucotricha* were mostly small flies from subfamily Mydaeinae (Muscidae) [56] that completely introduced their bodies into the small tubular flowers while pollen was attached onto the head and thorax (nototribic pollen deposition). It is known that larvae of some of these species are agricultural pests in South America, as secondary invaders of spoiled fruit and vegetables; thus, rotting vegetation, rather than or in addition to herbivore dung, may serve as a model in deceptive pollination of *J. leucotricha*. Interestingly, over 60% (by TIC peak area) of floral emissions of *J. leucotricha* can be accounted for by four isomers of reduced monoterpenoids, especially two isomers of dehydroxy-linalool oxide (herboxide), which is common in essential oils and wines but is rare in floral scents [101]. These abundant compounds were unique to *J. leucotricha* in our data set. As observed for *J. laciniata*, overnight equilibration of cut flowers of *J. leucotricha* expanded the complexity of volatile compounds collected on SPE micro traps for TD-GC-MS from 10 (Table S3) to 36 VOCs, including seven aliphatic ketones and a series of novel unknowns (Table S5). Headspace samples collected for 10 or 15 min did not differ substantially, but the 20 min collection crossed a threshold of detection for two long-chain aldehydes, two thioesters found in *J. sativa* (S-methyl thiopropanoate, S-methyl thiobutanoate) and a series of VOCs with a distinctive base peak of *m*/*z* 97, suggesting a cyclohexanone or dimethyl furanone skeleton (Table S5). The unusual floral scent composition of *J. leucotricha*, conforming to global expectations of herbivore dung mimicry, provides the clearest example of a divergent niche among fly-pollinated Andean clade species of *Jaborosa*. It remains unclear whether thioesters, which dominated the floral headspace of *J. sativa* but were present only at trace levels, and only under certain conditions, in the headspace of *J. laciniata* and *J. leucotricha*, contribute to fly attraction in field settings.

### 3.4. Jaborosa reflexa: Transition to Generalized Pollination in the Andes

Not all species of *Jaborosa* have specialized pollination systems relevant to specific syndromes. Among the five *Jaborosa* species sampled from the Andean clade, *J. reflexa* is the only species with a generalized pollination system, producing nearly glabrous flowers that are polymorphic for color and are visited by small flies (Syrphidae), medium size flies (Sarcophagidae) and bees (Halictidae) [56]. Accordingly, flowers of *J. reflexa* produce a scent profile containing more conventional aromatic and isoprenoid volatiles common to flowering plants with diverse pollination systems [1]. These compounds are similar to those of the hawkmoth-pollinated *Jaborosa* species, although they are emitted in much lower quantities in *J. reflexa* (Tables S2 and S3). Furthermore, *J. reflexa* samples in our survey were collected from three different populations, all of which produce linalool and (E)-DMNT (e.g., Río Chico) but which otherwise varied in the presence of methyl benzoate and farnesenes (El Trebol) or long-chain alkenals and benzyl benzoate (La Leona).

Ancestral state reconstruction suggests that *J. reflexa* may have diverged recently from a saprophilous fly-pollinated ancestor of the Andean clade [55]. Although the origins of this divergence are unclear, extant populations of *J. reflexa* overlap in both ENM dimensions and actual geographic ranges with *J. laciniata* and *J. leucotricha* (Figure S4), which are pollinated by distinct guilds of flies [56]. The generalized pollination of *J. reflexa* may facilitate coexistence with other *Jaborosa* species through reduced competition for pollinators. Results of our previous studies with other *Jaborosa* species make it clear that the experimental restoration of oligosulfides (DMDS, DMTS) to flowers of *J. reflexa* would instantly attract carrion flies to augmented flowers in their Andean habitat (see [58]).

The closest relative to *J. reflexa* in our study, *J. laciniata*, also emits some conventional volatiles (e.g., linalool, methyl benzoate), which we predict may be shared with a sister species (*Jaborosa kurtzii* Hunz. and Barboza) whose floral biology also suggests generalized pollination [55,56]. If *J. reflexa* represents a transition from a fly-pollinated ancestry to a more generalized pollination system, we hypothesize that this might have been accomplished through the loss of glandular trichomes responsible for emissions of S- and/or N-bearing volatiles in a putatively fly-pollinated ancestor. This inference is based on the observation that S-volatiles are produced by trichome-covered immature flowers and opening buds of *J. rotacea* [57] and *J. sativa* (Table S4), whereas the flowers of *J. reflexa* and the closely related *J. kurtzii* are nearly glabrous (see Figure 3 in [55]).

### 3.5. Environmental Influence on Floral VOCs and Pollinator Climate

Environmental variables that contributed the most to the ENMs of the two *Jaborosa* clades provide candidate factors to explain the differences observed in scent profiles and pollination modes. One challenge inherent to niche modeling approaches is the need for caution when using current climatic or physiological phenomena to interpret historical or paleo-ecological inferences emerging from such models. For example, it is known that drought may affect total VOCs released per flower and its chemical composition as a plastic response in real (ecological) time, and that the magnitude of the response is species-specific [37,102,103]. It is less well understood whether plants adapted to drought (or other environmental stressors) tend to produce different blends of volatile compounds constitutively, for reasons ranging from water balance to pleiotropic responses to plant stress (e.g., anthocyanin pigments). For example, one study [36] showed that exposure to different levels of ozone decreased the relative concentrations of monoterpenes and anisaldehyde, but increased the release of benzaldehyde from flowers of *Brassica nigra* (L.) W.D.J. Koch. Such changes in blend ratios are likely to impact visitation by both mutualists and antagonists [9,104]. Similarly, higher temperatures generally increase floral VOC emissions due to biosynthetic enzyme kinetics [105,106], suggesting a general enhancement of floral scent under warmer and/or drier conditions. We speculate that plants expanding into habitats in which heat, desiccation, UV irradiance, wind and changes in relative humidity represent novel selective pressures, might respond in ways that either directly or indirectly alter their potential for volatile biosynthesis and emissions. *Jaborosa* species in the Andean clade include plants whose entire surfaces (leaf, stem and flower) are covered with several classes of glandular trichomes, whose primary functions may be to protect against desiccation, UV damage and/or herbivore attack. Such trichomes may represent exaptations for shifts in volatile emission and blend composition in Andean *Jaborosa* species.

Our findings suggest that diversification in floral scent could have occurred in concert with shifts in environmental suitability accompanied by geographical differences in pollinator availability, specifically, the reduced abundance of hawkmoths. Diurnal range variation of temperature, the most important variable explaining the environmental suitability of Andean clade species, is also known to affect insect locomotion and physiology [107]. Temperature also contributes to diel differences in floral scent emission profiles [40], thus directly influencing the floral signals that attract both flies and hawkmoths [71,108]. The harsh environmental conditions in high elevation Andean habitats (low temperatures, short growing seasons, and strong winds) place constraints on potential visitation by nocturnal hawkmoths because they are endothermic insects that rarely fly above 1300 m in Subtropical Argentina [109]. Hawkmoth diversity is higher in subtropical lowland areas where ambient temperatures at dusk are moderate [43,81,110]. The oxygenated sesquiterpenes or aromatic VOCs identified from the hawkmoth-pollinated Subtropical Lowland clade species of *Jaborosa* were previously shown to be targets of phenotypic selection in lowland but not in mountain habitats for *Gymnadenia odoratissima*, a European orchid species [33]. Altitude may be indirectly affecting scent emissions through its effect on the occurrence of hawkmoths, pollinators that are globally constrained to lowland areas.

Pollination by saprophilous flies becomes substantial in the high-mountain Andes, where pollinator limitation strongly constrains the reproductive success of self-incompatible plant species [111,112]. In particular, species of the flesh-fly genus *Microcerella* Macquart, recorded as pollinators of the Patagonian species *J. magellanica* and *J. reflexa*, show greater diversity and higher abundances in arid and high-altitude environments of South America [113]. Saprophilous fly-pollinated species of *Jaborosa* are restricted to regions where species pollinated by long-tongued hawkmoths are absent (neither beyond 40° South latitude nor above 3000 m in the Andes). This pattern, combined with the inferred directions of evolutionary transitions in *Jaborosa* (hawkmoth to fly pollination, warm, humid lowland to cold, arid highland habitats), is consistent with the concept of pollinator climates as outlined by Grant and Grant [42] (see [28,29]).

## 4. Materials and Methods

### 4.1. Study System

The nightshade genus *Jaborosa* comprises 22 species endemic to southern South America and exhibits remarkable interspecific variation in floral traits. Three pollination modes have been reported in the genus: hawkmoths (and smaller moths), saprophilous flies, and generalized pollination by small insects [56]. Previous phylogenetic analyses recovered two strongly supported clades: a “Subtropical Lowland clade” including the three species with sphingophilous (hawkmoth-pollinated) flowers inhabiting lowland areas, and an “Andean clade”, including the remaining species of *Jaborosa*, mainly distributed across the Monte and Prepuna deserts, high mountain Andes and Patagonian steppe of temperate South America [55]. Here we studied the flower scent in eight focal *Jaborosa* species (Table S7). The topology of the phylogenetic tree used in subsequent analyses is a maximum clade credibility (MCC) tree from [55] based on four plastid markers and pruned to show only the studied taxa using the *phytools* package [114] of R 4.0.2 software [115].

### 4.2. Ecological Niche Modelling in Geographical Space

We obtained a total of 344 precise geographical coordinates of presence for 21 *Jaborosa* species, 289 for the Andean clade and 55 for the Lowland clade in the field or from vouchers deposited at CORD herbarium (coordinates data available upon request to corresponding author). From this dataset, we retained 196 points for the 18 species of the Andean clade, and 43 for the three species of the Lowland clade to prevent any bias due to high point density derived mainly from oversampling of more accessible areas. The model was manually calibrated in QGIS v. 3.10.4 [116]; 70% of the occurrence dataset was used for calibration and 30 % for validation. We delimited the extent of the modelling area (*“M”* sensu [117]) following topographic criteria, under the hypothesis that each clade distribution was constrained by the altitude.

To adjust ecological niche models (ENMs; [118]), we used 19 bioclimatic variables (2000s decade; 2.5 arcmin resolution) downloaded from the Dryad Digital Repository at http://datadryad.org/resource/doi:10.5061/dryad.s2v81 (accessed on 20 October 2020), which were obtained from a global set of satellite-based bioclimatic variables (MERRAclim; [119]), and the Hydro-1K (HYDRO1k Elevation Derivative Database, https://doi.org/10.5066/F77P8WN0) (accessed on 20 October 2020), a topographical database downloaded from the US Geological Survey (2001; http://lpdaac.usgs.gov (accessed on 20 October 2020). In the calibration, we discarded highly correlated environmental layers (Pearson > 0.9) which also showed a lower gain, according to the permutation importance and jackknife test.

We tested a total of six different modelling algorithms: BIOCLIM [120], Domain [121], and MaxEnt [122] using the R package *dismo* [123]; generalized linear model using *glm* (see [124]); random forest [125] with *randomForest* [126]; and support vector machines [127] with *kernlab* [128]. In particular, MaxEnt ENM was evaluated with the R package *ENMeval* [129] to test a set of predictor variables (“feature types”) together with different multiplier regularization values, totaling 48 models, of which the three with the lowest AICc values and lowest omission rates were chosen, and then modeled in MaxEnt v.3.3.3 [122]. We validated all algorithms using 30% of the presence points and ten absence points for each clade in NicheToolBox using partial receiver operating characteristic (ROC) and threshold-dependent tests [130]. Suitability and binary maps of all models were generated using the smallest suitability value from the species presence points as the threshold value.

Considering the validation metrics, we selected for the Andean Clade: MaxEnt (feature types: Linear and Quadratic, regularization multiplier: 3) and Bioclim algorithms; and for the Subtropical Lowland Clade: Bioclim and support vector machines algorithms. In order to capture most of the variation in the environmental data among *Jaborosa* species we conducted a principal component analysis (PCA) by using the *princomp()* function in R software. We included the environmental variables that most contributed (adding up at least 90%) to the ENMs of both clades and extracted the first principal component (PC1) that captured environmental variation. These PC1 scores were used in subsequent analyses.

### 4.3. Floral Scent Collection and VOCs Identification

We characterized the chemical composition of flower scent in eight focal *Jaborosa* species (Table S3) that encompass the three pollination modes present in the genus: *J. integrifolia*, *J. odonelliana* and *J. runcinata* by nocturnal hawkmoths (Sphingidae); *J. leucotricha*, *J. laciniata*, *J. sativa* and *J. rotacea* by saprophilous flies (Anthomyiidae, Calliphoridae, Muscidae, Sarcophagidae); and *J. reflexa* with generalized pollination by small insects (Syrphidae, Sarcophagidae and Halictidae). With the methods available to us (detailed below), we failed to detect any floral VOCs in two additional *Jaborosa* species: *Jaborosa bergii* Hieron. with a faint floral scent (unknown pollinators) and *J. magellanica* with a foul floral scent (saprophilous fly-pollinated).

Floral volatiles were sampled opportunistically from natural populations in Argentina from 2008 to 2014 and were analyzed by gas chromatography–mass spectrometry (GC-MS) using three different but complementary methods: dynamic headspace desorbed with solvent, solid-phase extraction (SPE) microtraps combined with thermal desorption and solid-phase micro extraction fibers (SPME).

Initially (2008–2010), we used dynamic headspace methods (see [13]) to collect scent from natural populations. We placed 4 L nylon resin oven bags (Reynolds Consumer Products, Inc., Lake Forest, IL, USA) over intact, blooming plants in the field to concentrate volatiles, then collected headspace air over glass Pasteur pipette traps with 10 mg of Porapak Q adsorbent (packed between plugs of silanized quartz wool) for 1 h, using 9V battery-operated PAS-5000 vacuum pumps (Spectrex, Inc., Redwood City, CA, USA) at a flow rate of 200 mL air/min. Trapped volatiles were eluted with 300 uL of GC-MS purity methylene chloride (2008) or hexane (2009–2010) (Sigma-Aldrich, Inc., St. Louis, MO, USA), concentrated to 75 μL (2008) or 50 μL (2009-2010) with a flow of gaseous N2, and 1 μL of each sample was injected in the GC-MS. A toluene dilution (5 μL of 0.03% toluene in hexane) was added to the concentrated samples as an internal standard [131]. Consistent with previous studies using similar methods [22,131], samples from hawkmoth-pollinated *Jaborosa* species produced chemical results that matched our olfactory perception (i.e., in emission rates and identified VOCs as character-bearing notes; see [65]). However, samples collected from taxa that we perceived as faint or foul scented yielded blank chromatograms using the dynamic headspace method.

Thus, in subsequent years (2011–2014) we employed another method, direct (solvent free) thermal desorption coupled with GC-MS, to characterize floral scent composition mostly from the Andean clade species. We constructed solid-phase extraction (SPE) microtraps that are loaded directly into a modified GC injection port liner for thermal desorption, by filling small cut glass capillaries (15 mm long, 0.2 mm internal diameter) either with 5 mg Tenax TA (60/80 mesh; for R.A. Raguso) or with a blend of 1.5 mg Tenax TA (60/80 mesh) and 1.5 mg Carbotrap B (for S. Dötterl) between plugs of silanized quartz wool (see [132]). Living flowers or entire plants were enclosed within bags constructed from sealed oven bag material (nylon resin; Reynolds, Inc.)) using an impulse heat sealer. The size of the headspace chambers varied according to the size and architecture of the flowers for each species (which vary greatly within the genus), always with the goal of minimizing headspace air volume [133]. When climatic conditions were harsh (e.g., high wind and frozen temperatures in the Andes), flowers of *J. laciniata* and *J. leucotricha* were cut and placed inside oven-clean glass vials (10–20 mL). Headspace air was allowed to equilibrate for 1 h, and volatiles were collected for 5 min (occasionally for longer times) using 9V battery-operated vacuum pumps (Spectrex, Inc.) set to 200 mL air/min flow rates. The microtraps containing headspace volatiles were stored within Teflon-capped glass autosampler vials (1.5 mL) without refrigeration in the field and then were kept at −20 °C in the laboratory until they were analyzed by GC-MS. Floral scent was collected from 2–6 individuals per *Jaborosa* species from field populations, with vegetative (n = 1) and ambient (n = 1) samples collected for each species as controls to identify and account for non-floral volatile artifacts present in the floral scent samples. Trapped samples were thermally desorbed for analysis by coupled GC-MS in two different laboratories, with comparable analytical systems (R.A. Raguso, Cornell University, USA; Shimadzu 2010+ GC-MS, and S. Dötterl, University of Bayreuth, Germany; Varian Saturn 2000), both using non-polar GC columns (stationary phase = 5% diphenyl, 95% polydimethyl siloxane).

Solid-phase microextraction fibers (SPME; Supelco, Inc., Bellefonte, PA, USA), combined with GC-MS, were used to characterize floral scent in the two species that we could cultivate in the greenhouse at Cornell University (*J. integrifolia* and *J. sativa*). We used two standard SPME fiber stationary phases (100 μm PDMS and 65 μm DVB/PDMS) to optimally adsorb different classes of VOCs expected from hawkmoth- and fly-pollinated flowers, as validated in previous studies [77,134]. SPME fibers were desorbed directly within the injection port of a gas chromatograph (GC17A) and analyzed using a quadrupole, EI mass spectrometer (QP-5000; Shimadzu Scientific Instruments, Inc., Columbia, MD, USA) as detailed by [57]. The number of collected samples using the three different methods is summarized in Supplementary Table S7.

Volatile compounds were tentatively identified using NIST, Wiley, and Adams mass spectral libraries and confirmed whenever possible through co-injection of authentic standards. For a subset of species analyzed using a polar GC column (stabilwax), the Kovats retention index of each compound was calculated through sample co-injection with n-alkane standards and then compared with published values (see http://www.pherobase.com/ (accessed on 31 March 2021) and http://www.flavornet.org/ (accessed on 31 March 2021) for compounds with similar mass spectra, as suggested by MS library search. Retention indices for the polar column, along with the 10 most abundant MS ion fragments, were listed for each compound that could not be (tentatively) identified in this way. 

### 4.4. Floral Scent Variation among Jaborosa Species Associated with Pollination Mode and Climate

Analyses to study the variation of floral scent among *Jaborosa* species were undertaken using the SPE dataset. Volatile compounds were grouped into seven biosynthetic chemical classes: aliphatics, aromatics, irregular terpenoids, monoterpenes, sesquiterpenes, nitrogenous compounds, and sulfurous compounds.

Two different approaches were used to analyze differences in scent composition among *Jaborosa* species. First, we constructed a plant–floral VOCs network considering floral scent compositions as connections between plant species and chemical compounds [135]. The matrix was constructed by averaging the relative percentage of floral VOCs per plant, and the *bipartite* package [136] of R software [115] was used to visualize the connectivity between *Jaborosa* species, pollination modes and floral VOCs. Second, we performed a non-metric multidimensional scaling (NMDS) based on pairwise Bray–Curtis dissimilarities. NMDS was conducted using the *metaMDS()* function of the *vegan* package [137] as implemented in the R software [115]. Calculations of stress were used to determine whether two-dimensional graphical representation of the NMDS coordinates was appropriate; the lower the stress value, the higher the quality of the ordination obtained [138]. Phylogenetic relationships among *Jaborosa* species were projected into the scent ordination space defined by the first two axes of the NMDS. Thus, the phylo-scent-space was constructed using the mean NMDS scores of each *Jaborosa* species and the MCC pruned tree using the *phylomorphospace()* function of the *phytools* package [114] in the R software [115]. Finally, to take into account the influence of the environmental context we projected environmental polygons and the six climatic variables that most contributed to the ENMs onto the phylo-scent ordination. This analysis was carried out with the *envfit()* function of the *vegan* package using 999 permutations. This function allowed us to fit environmental vectors onto the NMDS phylo-scent ordination. The projections of points onto vectors have maximum correlation with corresponding environmental variables.

### 4.5. Evolution of Floral Scent across Environments

To test whether evolutionary change in scent composition was concomitant with change in environmental niche occupancy, we carried out phylogenetic generalized least squares (PGLS) correlations between scent axes (i.e., NMDS1 and NMDS2 axes from Figure 3) and environmental axes (i.e., PC1 and PC2 axes from the environmental PCA). Two evolutionary models were fitted for correlated evolution: Brownian Motion (BM, [139]) and Ornstein–Uhlenbeck (OU; [140]). The OU model was chosen because it was identified by AIC as the best fit (Table S6). Analyses were carried out with functions *gls* of the *nlme* package [141] of R software [115].

## 5. Conclusions

Our work shed light on the potential causes of flower diversification in *Jaborosa*, complementing our previous finding of differences in sensory aspects of corolla color evolution between clades [56]). One important conclusion is that comparative studies such as ours are limited by enormous challenges—remote sampling, unstandardized methods, species refractory to cultivation—that are not often addressed in the literature. The use of a combined approach based on complementary modes of chemical analysis, multivariate analysis of the resulting scent profiles, ENMs to estimate environmental suitability areas, ancestral reconstruction of floral VOCs and pollination modes and the phylogenetic comparative method allowed us to conclude that the ability of *Jaborosa* plants to colonize newly formed environments during Andean orogeny and the ecological changes that followed were concomitant with significative changes in flower scent composition, several of which are known to trigger specific responses in nocturnal nectar-feeding hawkmoths and diurnal saprophilous flies. Selective forces exerted by specific groups of pollinators and geoclimatic variables that directly or indirectly shape flower traits (scent, color and shape) combine to explain the phenotypic differences we observed across the geographical range of this endemic South American genus.

## Supplementary Materials

The following are available online at https://www.mdpi.com/article/10.3390/plants10081512/s1, Figure S1: Non-metric multidimensional scaling (NMDS) of the floral scent profiles of Jaborosa sativa vary-ing the number and fresh mass of whole and dissected flowers (Supplementary Table S4). Figure S2: Metabolic series of rare floral scent compounds in *Jaborosa*. A. Sulfur metabolism in flowers of *J. sativa*. B. Nitrogen metabolism in flowers of *J. laciniata.* Figure S3: Non-metric multidimensional scaling (NMDS) shown floral scent samples that are shown averaged per species in Figure 3. White circles show *Jaborosa* samples (*J. runcinata*: 1–2, *J. integrifolia*: 3–4, *J. odonelliana*: 5–6, *J. reflexa*: 7–16, *J. sativa*: 17–18, *J. rotacea*: 19–20, *J. leucotricha:* 21–22, *J. laciniata*: 23–25), and red crosses the floral scent VOCs from Table S3. Figure S4: Environmental niche of *Jaborosa reflexa* and co-existing *Jaborosa* species. Table S1. Permutation importance value (%) of the bioclimatic variables used for the ecological niche models (ENMs) of the Andean and the Subtropical Lowland clades of *Jaborosa*. Table S2. Floral scent composition and emission rates from single flowers of *Jaborosa integrifolia* and *J. runcinata*, collected using dynamic headspace methods and analyzed via GC-MS. Table S3. Floral scent composition (presence, relative %) for eight species of *Jaborosa*, collected using solid-phase extraction micro-traps, combined with thermal desorption-GC-MS. Table S4. Intensive study of floral volatiles from *Jaborosa sativa* using SPME-GC-MS with a polar (EC-Wax) GC column, varying the number and fresh mass of whole and dissected flowers. Table S5. Incremental increase in sampling time for cut flowers of *Jaborosa leucotricha*, collected and analyzed using TD-GC-MS on a non-polar (DB-5) GC column. Table S6. Phylogenetic generalized least squares (PGLS) correlations between scent axes (NMDS1 and NMDS2 axes from Figure 3) and environmental axes (PC1 and PC2 axes from the environmental PCA). Two evolutionary models were fitted for correlated evolution: Brownian motion (BM, [139]) and Ornstein–Uhlenbeck (OU, Hansen, 1997). Table S7. Provenance data for the eight Jaborosa species studied. We provide information about floral scent collection method (SPE: Solid-phase extraction microtraps combined with thermal de-sorption, SPME: Solid-phase micro extraction fibers, Solvent: dynamic headspace desorbed with solvent), human perception of floral scent and pollination mode. Detailed information about pollinators’ identity is provided in [51] (Supplementary Table S7).
