# Supplementary material for: Floral Scent Evolution in the Genus Jaborosa (Solanaceae): Influence of Ecological and Environmental Factors
Source: Plants (Basel). 2021 Jul 23;10(8):1512. doi: 10.3390/plants10081512 (PMC8398055; doi:10.3390/plants10081512)
Supplement: Supplementary file 1 [file Figure S1.pdf › Figure S1.pdf]

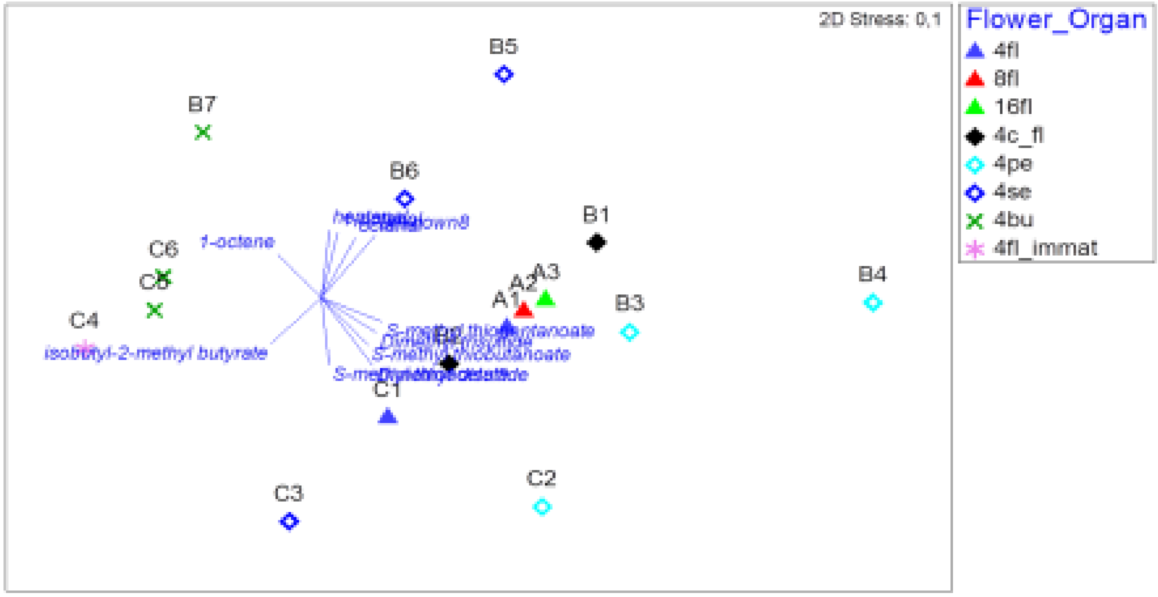

Figure S1: Non-metric multidimensional scaling (NMDS) of the floral scent profiles of *Jaborosa* sativa varying the number and fresh mass of whole and dissected flowers (Supplementary Table S4).
