# Supplementary material for: Floral Scent Evolution in the Genus Jaborosa (Solanaceae): Influence of Ecological and Environmental Factors
Source: Plants (Basel). 2021 Jul 23;10(8):1512. doi: 10.3390/plants10081512 (PMC8398055; doi:10.3390/plants10081512)
Supplement: Supplementary file 1 [file plants-10-01512-s001.zip › Figure S2.pdf]

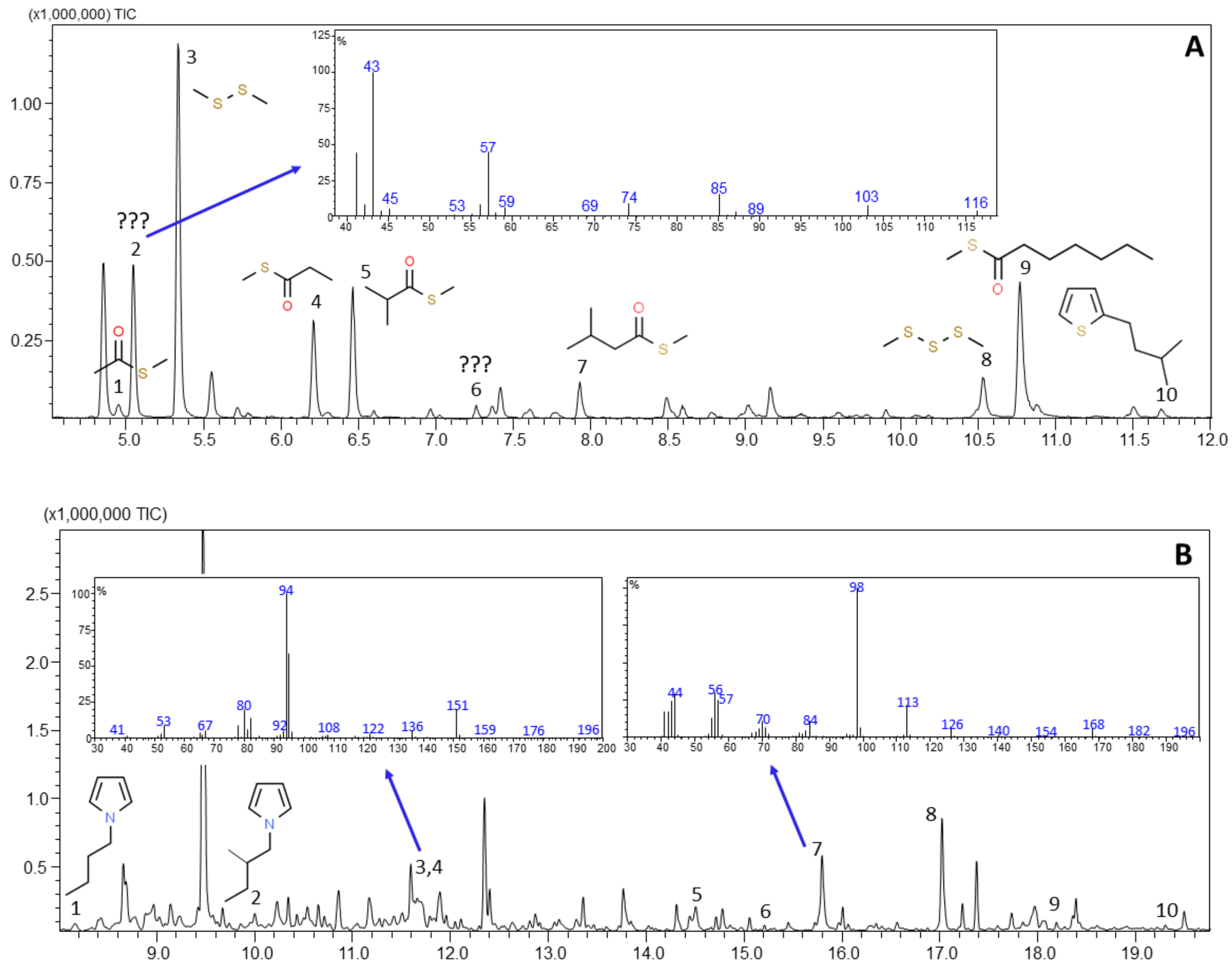

Figure S2: Metabolic series of rare floral scent compounds in *Jaborosa*. Total ion chromatograms (TIC) show peak traces in units of 1M counts (vertical axis) and retention time, minutes (horizontal axis). Inset mass spectra (MS) show 70eV quadrupole MS ion fragments with the base peak scaled to 100% (vertical axis) and fragments arrayed horizontally by m/z. Compound identifications are summarized in Tables S3 and S4. Molecular structures are shown courtesy of an open-source chemical database (http://www.chemspider.com/).

A. Sulfur metabolism in flowers of *J. sativa*, collected by SPME and analyzed on a non-polar (stabilwax) GC column. Numbered peaks include thioesters (1,2,4-7,9,10), oligosulfides (3,8) and a thiophene (10). The mass spectrum associated with peak 2 did not produce clear matches in NIST, Wiley or Adams MS libraries but suggests an isopropyl thioester.

B. Nitrogen metabolism in flowers *J. laciniata*, collected by SPE and analyzed on a polar (DB-5) GC column. Numbered peaks include pyrroles (1,2 and likely 3; left inset MS) and a homologous series of unknowns (4-10) with a common base peak (m/z = 98) suggestive of a methyl-piperidine ion fragment and smaller fragments in units of 14 m/z, indicating alkyl groups of varying chain length (right inset MS, from peak 7).
