# Supplementary material for: Floral Scent Evolution in the Genus Jaborosa (Solanaceae): Influence of Ecological and Environmental Factors
Source: Plants (Basel). 2021 Jul 23;10(8):1512. doi: 10.3390/plants10081512 (PMC8398055; doi:10.3390/plants10081512)
Supplement: Supplementary file 1 [file plants-10-01512-s001.zip › Figure S3.pdf]

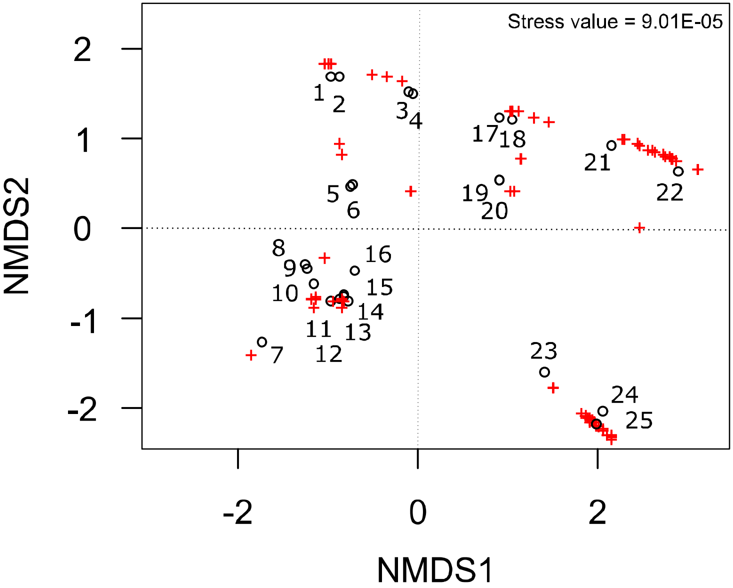

Figure S3: Non-metric Multidimensional Scaling (NMDS) shown floral scent samples that are shown averaged per species in Figure 3. White circles show *Jaborosa* samples (*J. runcinata*: 1-2, *J. integrifolia*: 3-4, *J. odonelliana*: 5-6, *J. reflexa*: 7-16, *J. sativa*: 17-18, *J. rotacea*: 19-20, *J. leucotricha*: 21-22, *J. laciniata*: 23-25), and red crosses the floral scent VOCs from Table S3.
