# Supplementary material for: Floral Scent Evolution in the Genus Jaborosa (Solanaceae): Influence of Ecological and Environmental Factors
Source: Plants (Basel). 2021 Jul 23;10(8):1512. doi: 10.3390/plants10081512 (PMC8398055; doi:10.3390/plants10081512)
Supplement: Supplementary file 1 [file plants-10-01512-s001.zip › Figure S4.pdf]

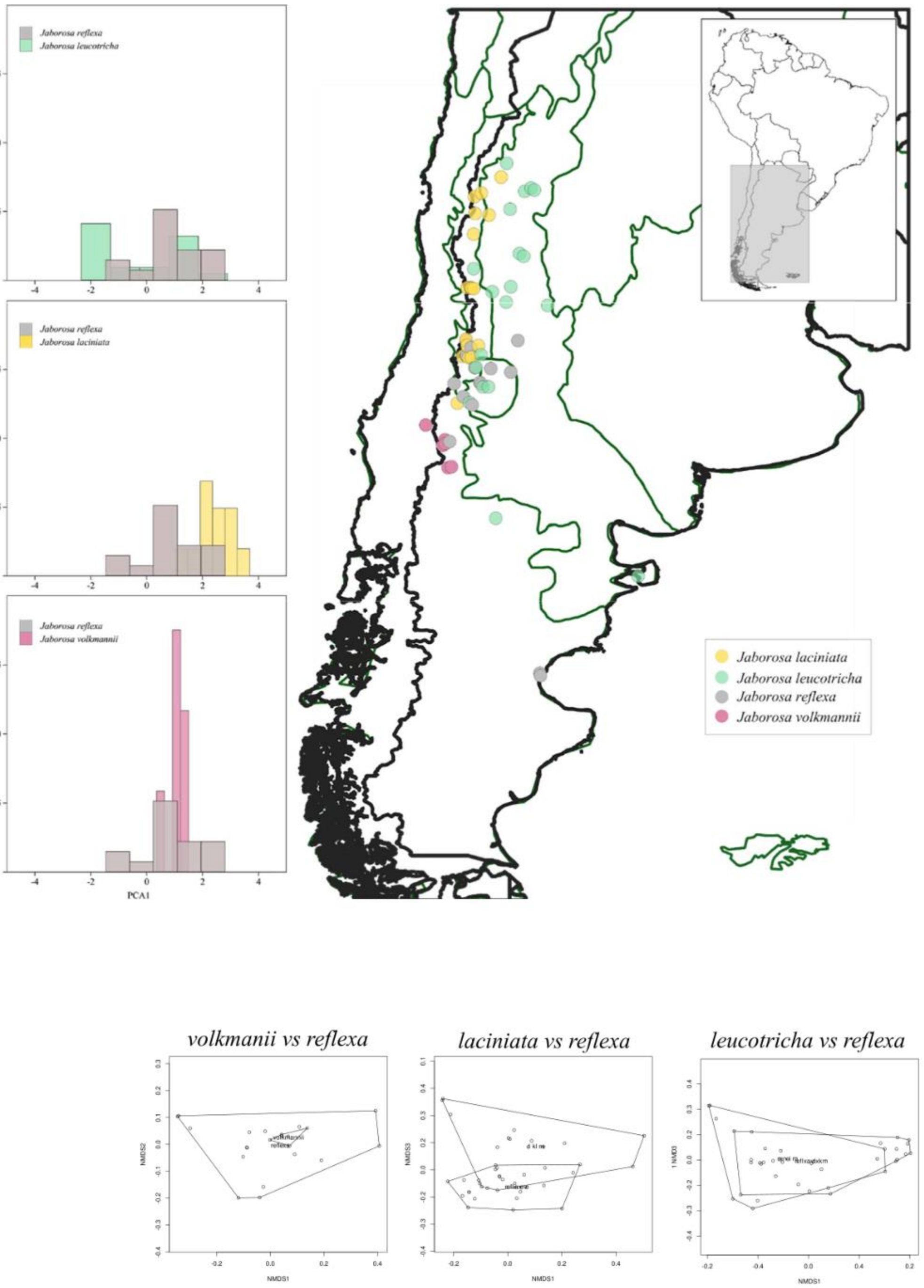

Figure S4: Environmental niche among *Jaborosa reflexa* and other three *Jaborosa* species that totally or partially overlap in their geographical distribution (*J. laciniata*, *J. leucotricha*, *J. volkmannii*). The environmental niche of *J. reflexa* significantly differed from *J. laciniata*, and partially overlapped with *J. leucotricha* and *J. volkmannii*.
