# Supplementary material for: Floral Scent Evolution in the Genus Jaborosa (Solanaceae): Influence of Ecological and Environmental Factors
Source: Plants (Basel). 2021 Jul 23;10(8):1512. doi: 10.3390/plants10081512 (PMC8398055; doi:10.3390/plants10081512)
Supplement: Supplementary file 1 [file plants-10-01512-s001.zip › Table S1.pdf]

Table S1. A. Permutation importance value (%) of the bioclimatic variables used for the Ecological Niche Models (ENMs) of the Andean and the Subtropical Lowland clades of *Jaborosa*.

**Bioclimatic variable**	**Permutation importance**
Andean clade	
mean diurnal range temperature	46.1
specific humidity seasonality	20.4
maximum temperature of the warmest month	15
temperature seasonality[2]	12.2
altitude	2.5
mean temperature of the least humid quarter	2.1
mean specific humidity of the coldest quarter	1.1
isothermality[1]	0.6
Subtropical Lowland clade	
altitude	72.6
specific humidity seasonality[3]	17.3
specific humidity of the most humid month	3.8
mean diurnal range temperature	3
specific humidity of the least humid month	2.1
isothermality[1]	1.3

[1]mean diurnal range temperature/temperature annual range * 100
[2]standard deviation *100
[3]coefficient of variation
