# Supplementary material for: Floral Scent Evolution in the Genus Jaborosa (Solanaceae): Influence of Ecological and Environmental Factors
Source: Plants (Basel). 2021 Jul 23;10(8):1512. doi: 10.3390/plants10081512 (PMC8398055; doi:10.3390/plants10081512)
Supplement: Supplementary file 1 [file plants-10-01512-s001.zip › Table S6.pdf]

Table S6. Association between environmental suitability and floral scent in *Jaborosa*. Phylogenetic generalized least squares (PGLS) correlations between scent axes (NMDS1 and NMDS2 axes from Fig. 3) and environmental axes (PC1 and PC2 axes from the environmental PCA). Two evolutionary models were fitted for correlated evolution: Brownian Motion (BM, Felsenstein, 1973) and Ornstein-Uhlenbeck (OU, Hansen, 1997).

Environmental axis	Scent axis	F-value	p-value	AIC	Model
PC1	NMDS1	0.35	0.5738	32.46	BM
		1.60	0.2524	33.79	OU
PC1	NMDS2	0.15	0.7144	32.73	BM
		6.86	**0.0396**	31.27	OU
PC2	NMDS1	0.00	0.9953	24.81	BM
		0.11	0.7511	24.85	OU
PC2	NMDS2	0.11	0.7523	24.67	BM
		0.17	0.6986	24.80	OU
DF:	6				
